# A flexible Raspberry Pi-based data logger platform for Modbus sensors with Ansible deployment

**DOI:** 10.1016/j.ohx.2026.e00814

**Published:** 2026-07-14

**Authors:** Leon Keim, Steffen Hägele, Vivien Langhans, Holger Class

**Affiliations:** Institute for Modelling Hydraulic and Environmental Systems, University of Stuttgart, 70569 Stuttgart, Germany

**Keywords:** Modbus, RS-485, Raspberry Pi, Environmental monitoring, Data logger, Ansible

## Abstract

This article presents LibrePiLogger, an open-source data logging platform based on the Raspberry Pi for environmental monitoring using Modbus sensors over RS-485. The system combines the AtmosPyre Python library for sensor communication with Ansible-based deployment automation, allowing researchers to deploy sensor networks by editing a single YAML inventory file. Two hardware configurations are described: a minimal setup using a Raspberry Pi Zero with an RS-485 HAT, and a maximal setup using a Raspberry Pi 4 with a USB-to-RS-485 converter. Currently implemented sensors include the Vaisala GMP252 for CO${}_{2}$ and the RadonTech AlphaTRACER for ^222^Rn, with new sensors requiring approximately 100 lines of Python following a provided driver template. Data is logged to timestamped CSV files with JSON metadata. The system has been deployed for continuous CO${}_{2}$ and ^222^Rn monitoring in a karst environment since spring 2025 and remains in active operation, demonstrating reliable long-term performance. All hardware designs, software, and deployment scripts are released under the GNU General Public License v3.0. Total hardware costs range from 54 to 63€ (excluding housing), depending on the configuration.

## Graphical abstract

**Figure:**
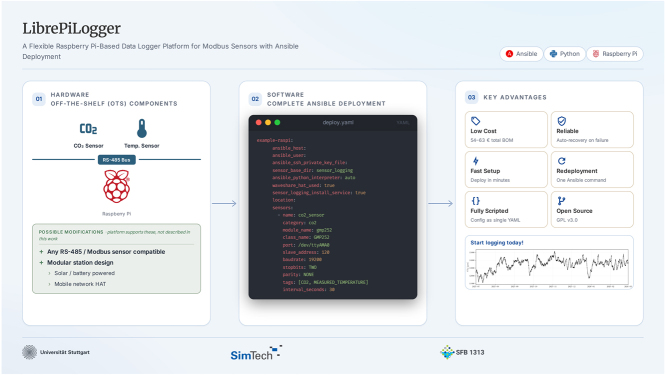

## Specifications table

**Table:** 

Hardware name	LibrePiLogger
Subject area	• Environmental, planetary and agricultural sciences
Hardware type	• Field measurements and sensors • Electrical engineering and computer science
Closest commercial analog	• Radon Scout, AlphaE or AlphaGUARD for radon monitoring • Vaisala handheld CO${}_{2}$ meter
Hardware license	GNU General Public License v3.0 or later
Software license	GNU General Public License v3.0 or later
Cost of hardware	54–63€ (excluding housing)
Source file repository	• Combined repository (hardware design files, software, and deployment scripts): https://doi.org/10.5281/zenodo.21257389 • AtmosPyre Software: – PyPI: https://pypi.org/project/atmospyre/ – Git: https://gitlab.com/libre-pilogger/atmospyre • Deployment Scripts: – Git: https://gitlab.com/libre-pilogger/senso-pi

## 1 Hardware in context

Long-term monitoring of gases such as CO${}_{2}$ and ^222^Rn in epigenic karst systems is essential for understanding site-specific transport dynamics, where dilution and accumulation of gases are governed by ventilation patterns and source terms; source strength is seasonally varying for CO${}_{2}$ but relatively constant for ^222^Rn. In many karst systems, much of the CO${}_{2}$ originates from microbial activity in the vadose zone, where multiphase gas–water transport takes place. CO${}_{2}$ dynamics, in particular, drive limestone and dolomite dissolution during karstification and strongly influence CO${}_{2}$ uptake at the air–water interface of stagnant water bodies through density-driven convective dissolution rather than turbulent mixing [1], [2], [3].

Continuous monitoring in remote, subterranean environments requires a robust, adaptable, and cost-efficient system capable of autonomous data acquisition, storage, and optional remote transmission. Numerous studies have reported CO${}_{2}$ and ^222^Rn measurement systems in various contexts, including occupational environments, outdoor field investigations, and subterranean karst systems. However, many of these works provide limited methodological transparency, describing only the sensors used while omitting details about the corresponding data logging system [4]. Others rely on commercially available data loggers or integrated devices, such as the Radon Scout, AlphaE or AlphaGUARD for radon monitoring, the Vaisala handheld CO${}_{2}$ meter [5], [6], or the Ahlborn data logger [7]. While these systems offer reliable performance, they are typically expensive and lack flexibility for adaptation to site-specific requirements or integration into customized monitoring networks.

Since cost factors and flexibility play an important role in making research studies feasible, several self-developed data logger systems have been described in the literature. Many of them are based on microcontrollers, such as PIC or Arduino. Kumar et al. [8] developed a PIC-based multi-channel data logger with integrated LCD display using serial RS-232 communication for wired data transfer to a host system, but without providing any open-source code. Brown et al. [9] and Levintal et al. [10] both proposed a low-cost CO${}_{2}$ monitoring system using microcontrollers like Arduino Uno and Adalogger on a printed circuit board, notable for its fully open-source hardware and software design. Kumar et al. [11] combined an Arduino board with a Raspberry Pi for IoT-based air quality monitoring, where the Pi acted as the major control node; however, the implementation details and code were not shared. These examples highlight the diversity of self-developed logger systems but also the lack of open and adaptable designs suitable for long-term environmental monitoring in complex field conditions.

The objective of our CO${}_{2}$ and ^222^Rn measurement setup, including a complete description and open-source code of the self-developed Raspberry Pi-based data logger, is to provide a cost-effective, flexible, and user-friendly solution. Compared to simple microcontroller-based systems, the Raspberry Pi offers significant advantages, including advanced networking and communication capabilities, larger storage capacity, and easier programmability, as it functions as a full-fledged computer running Python on a Linux platform. This combination allows for seamless integration of multiple sensors, reliable long-term data acquisition, and remote data access, making it particularly suitable for challenging environments such as underground karst systems.

## 2 Hardware description

The hardware system consists of commercial off-the-shelf components: Raspberry Pi units (Raspberry Pi Zero or higher), RS-485-to-USB adapters or RS-485 HATs, power supplies, and standard wiring assembled following a basic wiring diagram. The key distinction from existing sensor logging solutions is the combination of the AtmosPyre Python library for Modbus communication with an Ansible-based deployment system. Configuration requires only editing a YAML inventory file with sensor parameters; the deployment script handles virtual environment setup, dependency installation, systemd service creation, and automatic data logging initialization. The system works with any Modbus sensor, though sensors must first be implemented as AtmosPyre drivers to benefit from single-line Ansible deployment. Currently implemented sensors include the Vaisala GMP252 (CO${}_{2}$) and RadonTech AlphaTRACER (^222^Rn), with additional sensors requiring approximately 100 lines of Python following the provided driver template. Data is written to CSV files with JSON metadata, providing direct compatibility with standard scientific analysis tools.

**Key advantages for researchers:**

• **Protocol-based universality**: Any RS-485 Modbus sensor can be integrated. Implemented sensors deploy with a single command; new sensors require a Python driver ( 100 lines) following documented patterns, after which they gain the same deployment capabilities.
• **Deployment speed**: Ansible automation handles configuration, dependency management, and service installation automatically. Networks can be replicated across sites or reconfigured for new experiments in minutes rather than hours.
• **Low cost**: Raspberry Pi Zero units start around €15. No proprietary software licenses are required.
• **Data format**: Timestamped CSV output works directly with Python, R, MATLAB, and spreadsheet software.
• **Transparency and extensibility**: Both hardware assembly and software operation are fully visible and modifiable. Researchers can add new sensors following clear templates.
• **Modification**: Raspberry Pis can be tailored to specific use cases. For example, they can be used for mobile internet connection via an additional HAT or for mobile power supply using a battery.

## 3 Design files summary

**Table:** 

Design filename	File type	Open source license	Location of the file
wiring_diagram_usb	pdf	GPL 3.0 or later	[12]
wiring_diagram_usb	svg	GPL 3.0 or later	[12]
wiring_diagram_hat	pdf	GPL 3.0 or later	[12]
wiring_diagram_hat	svg	GPL 3.0 or later	[12]

There are two exemplary setups presented in this article: a minimal setup using an RS-485 HAT with a Raspberry Pi Zero, and a maximal setup using an RS-485-to-USB converter with a Raspberry Pi 4. The wiring diagrams of these setups are available in SVG and PDF respectively.

## 4 Bill of materials summary

In the following, two setups are showcased. The first is the minimal HAT setup using a Raspberry Pi Zero, while the second is the maximal USB converter setup using a Raspberry Pi 4 for greater flexibility. Standard items such as wires and clamps are not explicitly listed below. Depending on the sensors of interest, a different power supply setup might be more suitable. However, the key components remain as listed below.

### 4.1 Minimal HAT setup

This configuration represents the most cost- and power-effective deployment.

**Table:** 

Designator	Component	Number	Cost per unit - currency	Total cost - currency	Source of materials	Material type
RPI-01	Raspberry Pi Zero WH	1	16.70 EUR	16.70 EUR	reichelt.de	Semiconductor

HAT-01	Raspberry Pi Zero Shield - RS-485 CAN HAT, MCP2515	1	12.70 EUR	12.70 EUR	reichelt.de	Semiconductor

PSU-02	MeanWell MDR-40-5 DIN-Rail Power Supply, 5 V 6 A	1	20.47 EUR	20.47 EUR	conrad.de	Semiconductor

SD-01	MicroSD Card 8 GB Class 10	1	3.95 EUR	3.95 EUR	reichelt.de	Semiconductor

**Total cost (excluding sensors):**						**53.82 EUR**

### 4.2 Maximal USB converter setup

**Fig. 1:**
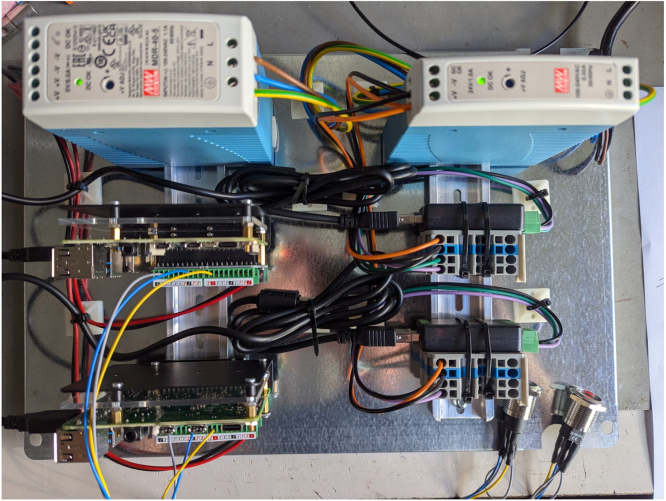
Top view of a setup with redundancy. Two Raspberry Pis simultaneously log CO${}_{2}$. It corresponds to the maximal setup but with a dedicated power supply.

This configuration provides more flexibility, suitable for laboratory environments or long-term monitoring stations where power consumption is not a primary concern. An exemplary build can be seen in Fig. 1.

**Table:** 

Designator	Component	Number	Cost per unit - currency	Total cost - currency	Source of materials	Material type
RPI-02	Raspberry Pi 4 Model B (4 GB RAM)	1	42.80 EUR	42.80 EUR	reichelt.de	Semiconductor

PSU-01	Official Raspberry Pi USB-C Power Supply 5.1 V 3 A	1	7.10 EUR	7.10 EUR	reichelt.de	Semiconductor

CONV-01	Waveshare Industrial USB to RS-485 Converter (CH343G)	1	8.50 EUR	8.50 EUR	berrybase.de	Semiconductor

SD-01	MicroSD Card 8 GB Class 10	1	3.95 EUR	3.95 EUR	reichelt.de	Semiconductor

**Total cost (excluding sensors):**						**62.35 EUR**

### 4.3 Housing

**Table:** 

Designator	Component	Number	Cost per unit - currency	Total cost - currency	Source of materials	Material type
MOUNT-01	Raspberry Pi DIN rail mounting clip	1	15.04 EUR	15.04 EUR	amazon.de	Polymer

RAIL-01	DIN rail 35 mm	2	7.50 EUR	15.00 EUR	reichelt.de	Metal

BOX-01	Control cabinet 300 x 400 x 170 mm (IP65)	1	43.55 EUR	43.55 EUR	reichelt.de	Metal

**Total cost:**						**73.59 EUR**

## 5 Build instructions

Both the minimal HAT setup and the maximal USB converter setup follow identical assembly principles, differing only in component selection; see the Design Files [12] or Fig. 2. The core concept involves mounting a Raspberry Pi with RS-485 communication capability alongside power supplies, then wiring sensor connections through screw terminals or directly to the RS-485 interface. Regardless of the setup chosen, when working with RS-485-based sensors and configuring them, there are a few things you need to know:

• The Raspberry Pi’s IP address and hostname
• The username you want to use on you Raspberry Pi
• The port the RS-485 signal is passed through
• RS-485 settings such as slave address, baud rate, parity, stop bits, byte size, and Modbus mode

Fig. 2Wiring diagrams for both LibrePiLogger hardware configurations. (a) Maximal setup: Raspberry Pi 4 with USB-to-RS-485 converter. (b) Minimal setup: Raspberry Pi Zero with RS-485 CAN HAT. Power supply, RS-485 interface, sensor connections, and ground routing are shown for each variant.Fig. 2

**(a):**
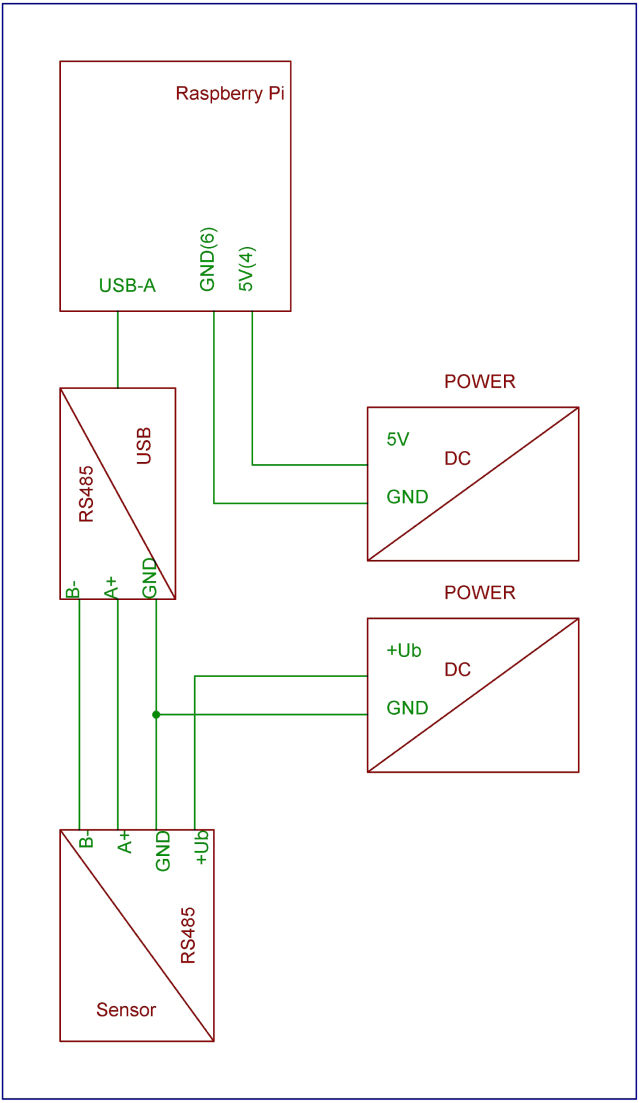
.

**(b):**
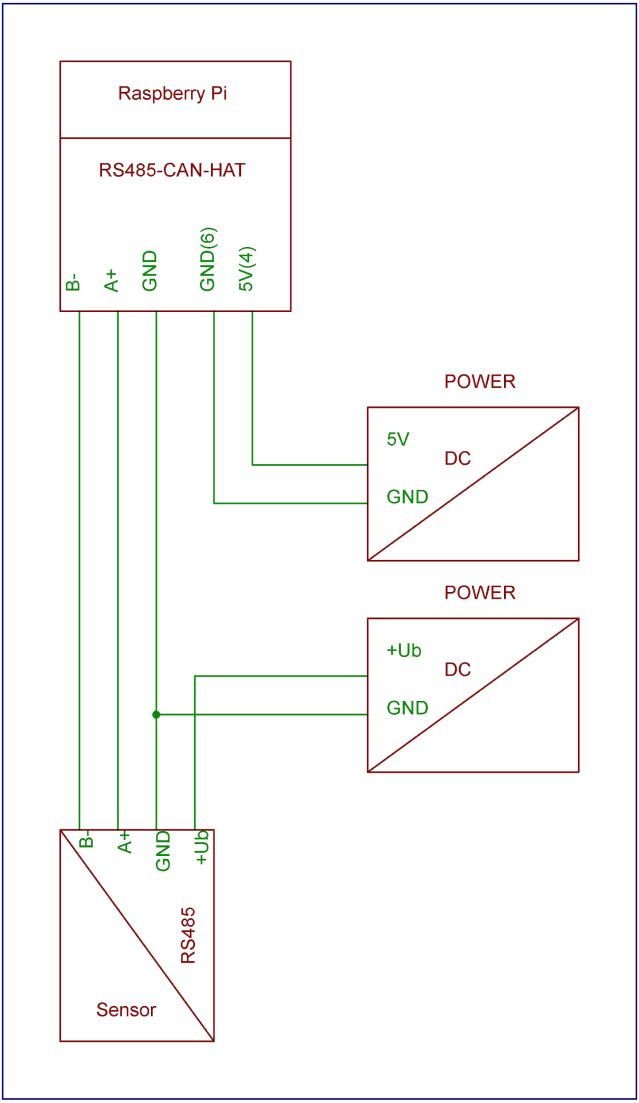
.

### 5.1 Safety considerations

#### Electrical safety

All assembly steps involving mains voltage (230V AC in Europe) must be performed with the system unplugged. If unfamiliar with electrical wiring, consult a qualified electrician for the mains connection. Do not operate the system with exposed mains voltage terminals.

#### Sensor power requirements

Verify sensor voltage requirements before connection. Most environmental sensors operate at 5–24V DC. Incorrect voltage can damage sensors permanently.

#### ESD protection

Raspberry Pi boards are sensitive to electrostatic discharge.

### 5.2 Pre-assembly preparation

#### Raspberry Pi setup

Install Raspberry Pi OS on the microSD card:

1. Download Raspberry Pi Imager from https://www.raspberrypi.com/software/ (we recommend using the latest Appimage)
2. Insert microSD card into computer
3. Select a suitable OS; the light versions are recommended.
4. Configure hostname, enable SSH, inject a public SSH key, and set username/password
5. Take note of the hostname, username and password and make sure you do not lose the SSH key
6. Write image to card
7. Insert card into Raspberry Pi
8. Find the local IP address of the Raspberry Pi
9. Test SSH connection

**Figure:**
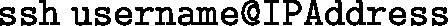

### 5.3 Mechanical assembly

#### Component mounting

For the minimal HAT setup: Attach the HAT to the Raspberry Pi Zero GPIO header before mounting. Ensure the HAT is firmly seated on all 40 pins.

For the maximal USB converter setup: Plug the USB-to-RS-485 converter into the Raspberry Pi 4 and secure it against bending the port.

### 5.4 Electrical connections

#### Raspberry Pi power connection

Here you have two options depending on your power source. You can either power the Raspberry Pi via the GPIO pins or use a USB port and the official power supply.

Power via GPIO pins:

1. +V output terminal on 5V power supply (PSU-02) → GPIO PIN 4
2. -V (GND) terminal on 5V power supply (PSU-02) → GPIO PIN 6

Power via USB: Plug the official Raspberry Pi USB power supply (PSU-01) directly into the Raspberry Pi. The type of USB will depend on the chosen Raspberry Pi model.

#### RS-485 wiring

RS-485 uses a differential pair (A and B lines) plus ground for communication. Sensors connect via three-wire or four-wire cable depending on the model.

1. Identify RS-485 terminals on the HAT or USB adapter (typically labeled A, B, GND)
2. Connect sensor wires (read the sensor manual carefully):
3. Sensor A (or D+) → HAT/USB adapter A
4. Sensor B (or D-) → HAT/USB adapter B
5. For the minimal HAT setup:
  • Sensor GND → Raspberry Pi GPIO GND
6. For the maximal USB converter setup:
  • Sensor GND → USB adapter GND

#### Sensor power connection

1. Verify sensor voltage requirement (typically 5–24V for environmental sensors)
2. Connect sensor power wires:
  • Sensor V+ → +V output terminal on sensor power supply
  • Sensor V- or GND → -V (GND) terminal on sensor power supply
3. Ensure polarity is correct; reversed polarity can damage sensors

#### Multiple sensor configurations

For laboratory setups monitoring multiple sensors simultaneously:

1. Connect all sensors to the same RS-485 bus (parallel connection of A, B, GND lines). For larger installations, a terminal resistor may be needed (standard value: 120 Ω).
2. Ensure each sensor has a unique Modbus slave address

#### Network connection

Depending on the network environment, contacting the system administrator may be necessary before proceeding.

Connect to the Raspberry Pi via SSH:

**Figure:**
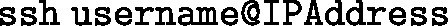

#### Deployment of sensors

Verify that the RS-485 interface is detected:

For the maximal USB converter setup:

**Figure:**
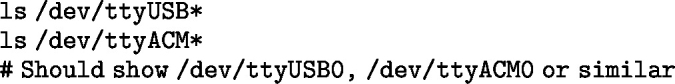

Take note of the port for later.

For the minimal HAT setup, the Ansible deployment handles the port automatically to use:

**Figure:**
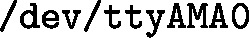

### 5.5 Software deployment

Software deployment is automated via Ansible.

Basic steps:

1. Install Ansible on the control computer:

**Figure:**
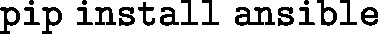
2. Clone the deployment repository at the latest stable release (check https://gitlab.com/libre-pilogger/senso-pi/-/releases for the most recent tag, currently v1.0.1):

**Figure:**
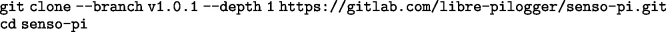
3. Install dependencies:

**Figure:**
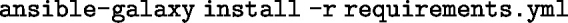
4. Create a custom inventory:

**Figure:**
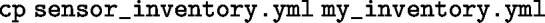
5. Modify the inventory for the specific setup:
  • IP address or hostname of the Raspberry Pi
  • The username you used when you set up the Raspberry Pi
  • Sensor type, serial port, Modbus address
  • Measurement tags, logging interval, and output directory
6. Full setup and deployment to the Raspberry Pi:

**Figure:**
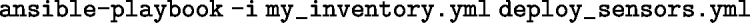
7. Redeployment to the Raspberry Pi:

**Figure:**
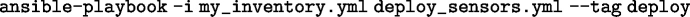

The deployment script automatically:

• Installs the AtmosPyre library and handles dependencies
• Generates a sensor-specific logging script
• Configures data output directories
• Sets up a systemd service (if requested)
• Starts data logging

### 5.6 Software design notes

#### Dependency management

AtmosPyre uses uv for environment management. Package dependencies are pinned via a committed lockfile regardless of architecture, ensuring identical library versions across deployments. The Python interpreter version is additionally fixed on 64-bit systems, where uv provides a standalone build independent of the system Python. Users may deploy a stable release tag for guaranteed reproducibility or track the development branch for the latest features, accepting reduced stability in return.

#### Bus scheduling and error handling

The scheduling architecture ensures reliable bus operation across multiple sensors. Sensors are polled sequentially in a single thread, preventing simultaneous RS-485 access. Each sensor is read with an individual timeout; a non-responding node is logged non-fatally as a timestamped entry in a daily error log, while polling continues for all remaining sensors and the failed node is retried on the next cycle.

### 5.7 Design alternatives and customization

#### Enclosure selection

The enclosures shown are weatherproof for outdoor deployment. For indoor laboratory use, simpler plastic project boxes reduce cost. Ensure adequate ventilation if operating in high ambient temperatures.

## 6 Operation instructions

### 6.1 Safety considerations

#### Electrical safety

Do not open the enclosure while connected to mains power. Disconnect power before any maintenance or inspection.

#### Environmental limits

Operation is limited by enclosure IP rating and component specifications.

#### Sensor handling

Follow manufacturer safety guidelines for specific sensors.

### 6.2 Normal operation

Once deployed, the system operates autonomously with no user interaction required. The systemd service ensures automatic startup after power interruption and continuous data logging at configured intervals.

#### System status check

Monitor system health via SSH (where [hostname] corresponds to the hostname configured in the Ansible inventory):

**Figure:**
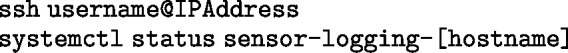

Active status indicates normal operation. Failed status requires log inspection:

**Figure:**
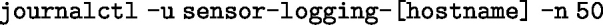

#### Data retrieval

Data files are organized by sensor and date in the sensor_logging/data/ directory. Each sensor creates a subdirectory containing daily CSV files with ISO 8601 timestamps.

Copy data to a local machine using SCP:

**Figure:**
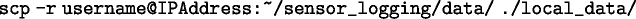

For regular automated backups, use rsync:

**Figure:**
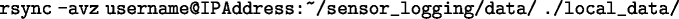

#### Stopping and removing the logging service

To stop the data logging service:

**Figure:**
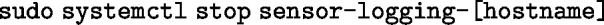

To prevent it from restarting after a reboot:

**Figure:**
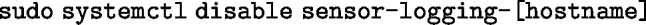

To fully remove the service file:

**Figure:**
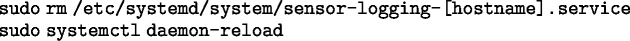

## 7 Validation and characterization

The system has been deployed since spring 2025 to measure CO${}_{2}$ and ^222^Rn in a karst cave environment characterized by high humidity (near saturation). Over the continuous monitoring period to date, all expected measurements were recorded successfully, with no gaps or corrupted records during system operation.

**Fig. 3:**
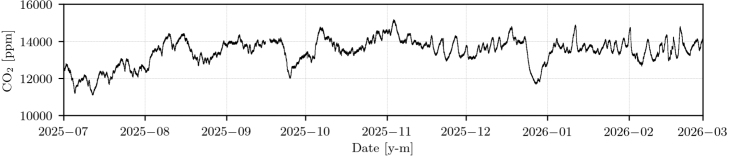
LibrePiLogger setup described in this article.

**Fig. 4:**
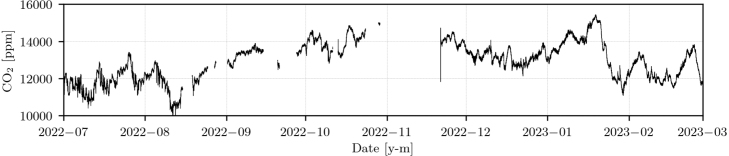
Legacy setup using commercial data loggers.

The proposed setup (Fig. 3) was validated against a legacy setup using commercial data loggers (Fig. 4), operated in parallel on the same site under identical environmental and power conditions. Both systems showed excellent agreement, with indistinguishable temporal trends and only a small systematic offset, remaining within expected sensor uncertainty throughout the parallel operation period. The legacy setup has been independently validated in a prior publication [2], where CO${}_{2}$ measurements in water using a permeable membrane showed very good agreement with independent laboratory analysis of water samples. Both setups rely on factory calibration; the observed offset is consistent with the expected drift between two instruments calibrated at different points in time.

The system service reliably recovers from power interruptions without user intervention. This behavior has been verified both during unplanned outages in the field (e.g. September 2025) and through repeated testing in the laboratory.

Once the Raspberry Pi OS is installed and the hardware is wired, software deployment of new sensors takes approximately 5–10 min using the Ansible playbook. This includes inventory configuration, dependency installation, and service activation. Users unfamiliar with the system should expect slightly longer times for initial setup.

The Ansible-based deployment also enables remote reconfiguration. During the field deployment, logging parameters such as the measurement interval were updated remotely without physical access to the device. As long as no hardware changes are required, the system can be fully reconfigured over the network.

The power draw of both hardware configurations was measured with one GMP252 sensor attached at a 30 s logging interval; values reflect the Raspberry Pi unit only and do not include sensor power consumption. As a ballpark figure, the GMP252 itself draws approximately 0.7 W. Table 1 summarizes the results.

**Table 1:** Measured power draw of both LibrePiLogger hardware configurations with one GMP252 sensor attached at a 30 s logging interval.

Raspberry Pi	OS	Avg. draw	Peak draw
4B	Pi OS Lite 64-bit	2.92 W	3.40 W
Zero W	Pi OS Lite 32-bit	1.24 W	1.36 W

### 7.1 Conclusions

LibrePiLogger has been demonstrated to operate continuously and autonomously in a challenging subterranean environment since spring 2025, with no gaps or corrupted records during system operation to date. The system recovers automatically from power interruptions without additional data loss and supports remote reconfiguration of logging parameters without physical access to the device. Multiple sensors can be logged simultaneously via the shared RS-485 bus, and a new sensor can be deployed in approximately 5–10 min after hardware assembly. Measurement accuracy and precision are determined by the sensor selected by the user; any RS-485 Modbus sensor is compatible.

The principal limitations of the platform are as follows. Remote operation requires network connectivity; without it, data must be retrieved manually via the microSD card. Adding a sensor not yet implemented in AtmosPyre requires writing a Python driver of approximately 100 lines, which assumes basic Python knowledge. RS-485 bus length is limited by the protocol, and the platform does not prescribe a specific power supply or enclosure, leaving these choices to the user.

## CRediT authorship contribution statement

**Leon Keim:** Writing – review & editing, Writing – original draft, Validation, Software, Conceptualization. **Steffen Hägele:** Resources, Methodology, Conceptualization. **Vivien Langhans:** Writing – review & editing, Writing – original draft, Data curation. **Holger Class:** Writing – review & editing, Writing – original draft, Supervision, Funding acquisition.

## Declaration of generative AI and AI-assisted technologies in the manuscript preparation process.

During the preparation of this work the authors used Claude (Anthropic) to support code cleaning and refactoring of the AtmosPyre library and deployment scripts in senso-pi, as well as to assist with manuscript writing and language editing. DeepL was additionally used for language and phrasing support. After using these tools, the authors reviewed and edited the content as needed and take full responsibility for the content of the published article.

## Declaration of competing interest

The authors declare that they have no known competing financial interests or personal relationships that could have appeared to influence the work reported in this paper.
